# Prevalence of sarcopenia and association with HIV infection in China elderly: An observational study

**DOI:** 10.1097/MD.0000000000038532

**Published:** 2024-06-28

**Authors:** Tiecheng Peng, Xin Jin, Chen Xiong, He Juan, Chen Juhai, Wang Longhang, Yiyi Wang

**Affiliations:** a School of Public Health, The Key Laboratory of Environmental Pollution Monitoring and Disease Control, Ministry of Education, Guizhou Medical University, Guiyang, China; b Department of Cardiology, Guiyang Public Health Treatment Center, Guiyang, Guizhou Province, China.

**Keywords:** association studies, elderly population, HIV, prevalence, sarcopenia

## Abstract

This article aims to analyze the prevalence of sarcopenia among the elderly in Guizhou Province, China, and its association with human immunodeficiency virus (HIV) infection. This cross-sectional study included 377 patients aged 60 and above in Guiyang Public Health Treatment Center from December 2022 to October 2023, including 231 patients in the community clinic and 146 HIV-infected individuals. According to the Asian Working Group for Sarcopenia 2019 Consensus to diagnose sarcopenia. Logistic regression was used to explore association between sarcopenia and HIV, and stratified by sex and age group. The prevalence of sarcopenia in the non-HIV infection elderly in Guizhou Province was 7.8% (21.3% in males and 5.5% in females), and the prevalence of sarcopenia in HIV-infected individuals was 29.5% (33.3% in males and 13.2% in females), with a statistically significant difference between HIV groups (χ^2^ = 30.946, *P* < .001). After control of gender, age, body mass index, body fat percentage, hypertension, diabetes, taking statins, smoking status, medium to high-intensity physical activity, whether childhood poverty, and parents died young, HIV infection was significantly associated with sarcopenia in the elderly (odds ratio = 4.635, 95% confidence interval  = 1.920–11.188, *P* = .001). The results of stratified regression were similar to the main results. The prevalence of sarcopenia in the elderly population in China was severe. HIV infection was a risk factor for sarcopenia. It is urgent to establish a prevention and treatment system for sarcopenia in the elderly population, especially for elderly HIV-infected male.

## 1. Introduction

Sarcopenia, commonly referred to as muscle loss syndrome, is a syndrome characterized by the progressive and systemic reduction of skeletal muscle mass and strength.^[1]^ Sarcopenia is highly prevalent among the elderly, potentially leading to adverse health outcomes such as falls, fractures and a significant decline in quality of life.^[2,3]^ In recent years, the rate of human immunodeficiency virus (HIV) infection among the elderly in China has rapidly increased as well.^[4]^ A meta-analysis showed that complications of HIV infection and extended exposure to highly active antiretroviral therapy (ART) were associated with accelerated aging and the loss of muscle mass and strength.^[5]^ This suggests that individuals infected with HIV may be at a higher risk of developing sarcopenia, creating a dual burden on the human body.^[6,7]^

Two studies conducted in India, which defined sarcopenia according to low muscle mass, indicated that the prevalence of sarcopenia was 40% among middle-aged male patients with HIV and 18% among premenopausal female patients with HIV.^[8,9]^ However, China currently lacks sarcopenia surveys based on the diagnostic and treatment expert consensus of sarcopenia published by the Asian Working Group for Sarcopenia (AWGS) in 2019,^[10]^ and research about the association between HIV infection and sarcopenia has not adequate.

This study aims to investigate the prevalence of sarcopenia among the elderly in Guizhou Province, China, and its association with HIV infection. The significance of this study lies in its potential to elucidate the association between sarcopenia and HIV infection in the elderly population of China, which could have profound implications for public health policies and the management of comorbidities in aging HIV-infected individuals. Furthermore, understanding the prevalence of sarcopenia in this demographic is crucial for developing targeted interventions to improve the quality of life and reduce healthcare burdens.

## 2. Subjects and methods

### 2.1. Study subjects

A cross-sectional study was conducted, collecting data from 377 elderly patients aged over 60 in the Guiyang City Public Health and Treatment Center from December 2022 to October 2023. Among these patients, 138 were from the community clinic, and 134 were HIV-infected individuals from the infectious disease outpatient clinic.

Inclusion criteria were as follows: age ≥ 60 years; possession of basic communication and comprehension abilities; provision of informed consent.

Exclusion criteria included: severe infection, acute cardiovascular or cerebrovascular events, major or medium surgery, trauma, or other stress conditions; need for walking assistance or presence of severe cognitive impairments that preclude cooperation with relevant measurements; internal placement of metal stents or pacemakers, or existence of other conditions that contraindicate bioelectrical impedance analysis or affect the accuracy of measurements; diagnosis of Parkinson syndrome, severe osteoarthritis affecting physical activity, or currently undergoing treatment for cancer; recent fractures causing mobility issues.

This study was reviewed and approved by the Ethical Committee of the Guiyang City Public Health and Treatment Center Hospital, with the approval number 202266. All enrolled patients provided informed consent.

### 2.2. Methods

#### 2.2.1. Diagnosis of sarcopenia

*Grip strength.* An electronic spring-type dynamograph (Guangdong Xiangshan Weighing Apparatus Group Company EH101) was utilized to measure grip strength. Participants stood with arms at their sides and palms facing inward, grasping the dynamograph with their dominant hand. They were instructed to squeeze the dynamograph with maximum effort for 3 seconds. The measurement was read and recorded. After a 30-second rest, the measurement was repeated once, and the maximum value was recorded as the dominant hand grip strength.

*SARC-F questionnaire.* This questionnaire includes 5 items: muscle strength, assistance in walking, rising from a chair, climbing stairs, and number of falls, with a total score of 10 points and a minimum of 0 points. A total score of ≥4 points is considered a high risk for sarcopenia.

*Muscle mass.* After measuring height with a standard stadiometer, bioelectrical impedance analysis (Inbody270, Biospace Company, Korea) was used to measure the appendicular skeletal muscle mass (ASM). The skeletal muscle index (SMI) was calculated with the formula: SMI = ASM(kg) ÷ height (m)^2^. In addition, participants’ body mass index (BMI) (kg/m^2^) and body fat percentage (%) were measured.

*Diagnosis of sarcopenia.* According to the Asian Working Group for Sarcopenia 2019 consensus (AWGS-2019) on the diagnosis of sarcopenia^[10]^:

SMI is measured using bioelectrical impedance analysis. If SMI < 7.0 kg/m^2^ in men or SMI < 5.7 kg/m^2^ in women, the participant was as reduced skeletal muscle mass.

If the grip strength of the upper limbs is <28 kg in male or <18 kg in female, the participant was diagnosed as reduced muscle strength.

SARC-F questionnaire score of ≥4 was defined as a decline in physical performance.

A diagnosis of sarcopenia was confirmed if criterion (1) is met, alongside any 1 of criteria (2) or (3).

#### 2.2.2. HIV diagnosis

The diagnosis of HIV was according to the diagnostic criteria for AIDS set by the Infectious Diseases Society of China Medical Association in the “Chinese Guidelines for Diagnosis and Treatment of AIDS (2021 Edition).”^[11]^ HIV infection is diagnosed following 1 of 2 criteria: Positive results from both an HIV antibody screening test and an HIV supplementary test, which includes either a positive antibody supplementary test or a positive qualitative nucleic acid test, or a quantitative nucleic acid test result >5000 copies/mL. In cases with a history of epidemiology or HIV-related clinical manifestations, HIV infection can be diagnosed if 2 HIV nucleic acid tests are positive. The diagnostic process consists of 2 main stages: the antibody screening test and the antibody supplementary test. The screening test includes various methods such as the enzyme-linked immunosorbent assay, immunofluorescence, and rapid tests. If the screening test is positive, it is followed by the antibody supplementary test for confirmation. In cases of ambiguity, a nucleic acid test or follow-up after 2 to 4 weeks can be performed. The supplementary test confirms the presence of HIV by detecting specific antibodies in the patient’s blood.

#### 2.2.3. Questionnaire survey

Professional survey personnel inquired about demographic information of the participants, including gender, age and residential situation. We also inquired participants’ chronic diseases and other health conditions (hypertension, diabetes, and whether taking statins and sleeping pills), smoking and drinking behavior (at least twice a week and for at least 3 years), average daily time of moderate to vigorous physical activities (medium to high-intensity physical activity, MVPA), and early life adverse experiences, such as exposure to childhood famine or poverty and parents died young (whether parents passed away before the participants’ age of eighteen).

### 2.3. Statistical methods

Statistical analyses were performed using SPSS 22.0 software (IBM, Chicago, IL, USA). Quantitative data were described as means ± standard deviations ($\bar{\chi }\pm s$), and categorical data were described as frequencies and percentages [n(%)]. The normality of the quantitative data was tested using the normal distribution test, and comparisons between groups were conducted using independent sample *t* tests. Chi-square (χ²) tests were used for the comparison of categorical data. The data is presented using bar charts, violin charts, and box plots to illustrate the distribution of variables. The violin chart extracts 50% of the samples as shake points. Variables with statistical significance were selected to build a multivariate Logistic regression model to analyze the association between sarcopenia and HIV infection. Multivariate logistic regression analysis was used to analyze the association between sarcopenia and HIV infection, adjusted for age group (<70, ≥70), gender, hypertension, diabetes, taking statins, BMI, smoking status, body fat percentage, MVPA, childhood poverty, and parents died young. Furthermore, stratification by age group and gender was performed to analyze the disparity in subgroups of association between sarcopenia and HIV infection.

## 3. Results

### 3.1. Prevalence of sarcopenia in the elderly in Guizhou Province, China

**Figure 1.**
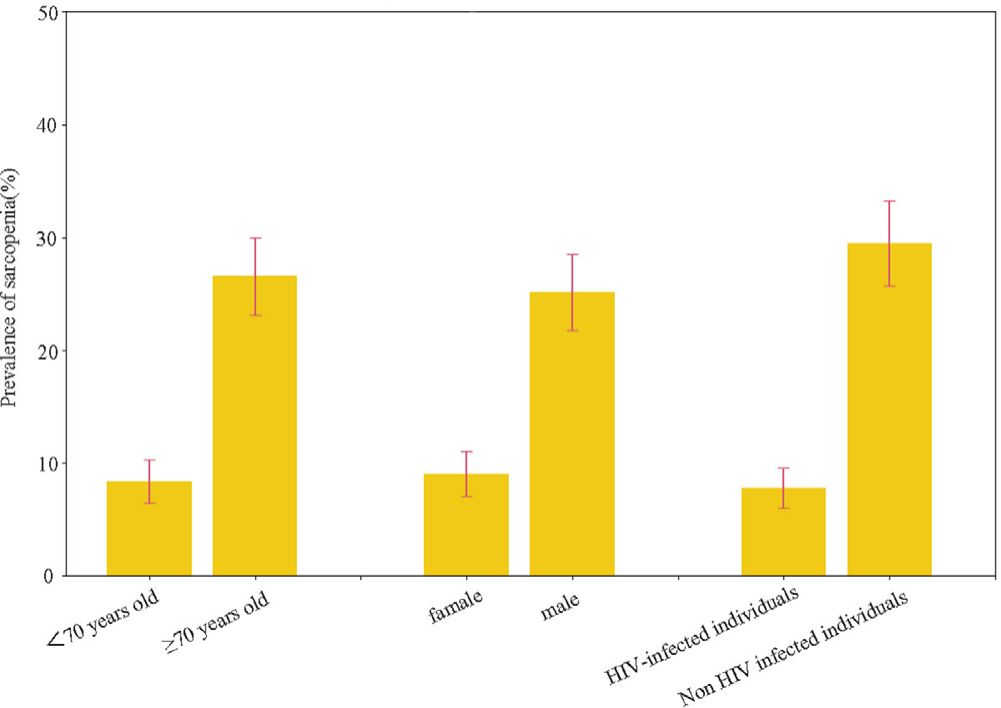
Prevalence of sarcopenia in different groups.

Basic information of the study participants was shown in Table 1. The average age of the participants was 69.89 (±6.73) years. One hundred sixty seven men were included (44.3%). The prevalence of sarcopenia among non-HIV-infected individuals in Guizhou Province was 7.8% (13.2% in males and 5.5% in females). The prevalence of sarcopenia among HIV-infected individuals was 29.5% (33.3% in males and 21.3% in females). There was a statistically significant difference of prevalence of sarcopenia between non-HIV-infected individuals and HIV-infected individuals (χ² = 30.946, *P* < .001) (Fig. 1).

**Table 1:** Prevalence and risk factors of sarcopenia in the elderly in Guizhou Province, China.

	Total population	Sarcopenia (n = 61)	Non sarcopenia (n = 316)	χ^2^/*t*	*P*
Age	69.89 (±6.73)	73.43 (±7.39)	69.20 (±6.39)	4.604	<.001
Height (cm)	158.82 (±7.98)	158.67 (±8.64)	158.85 (±7.87)	0.163	0.87
Weight (kg)	59.92 (±10.34)	52.86 (±7.58)	61.28 (±10.26)	6.072	<.001
BMI (kg/m^2^)	23.72 (±3.50)	21.01 (±2.61)	24.24 (±3.41)	7.010	<.001
Body fat rate (%)	30.80 (±7.58)	27.66 (±6.40)	31.41 (±7.65)	3.589	<.001
Sex					
Male	167	42 (25.1%)	125 (74.9%)	17.784	<.001
Female	210	19 (9.0%)	191 (91.0%)		
HIV infection					
Yes	146	43 (29.5%)	103 (70.5%)	30.946	<.001
No	231	18 (7.8%)	213 (92.2%)		
Diabetes					
Yes	92	8 (8.7%)	84 (91.3%)	5.027	.03
No	285	53 (18.6%)	232 (81.4%)		
Hypertension					
Yes	167	15 (9.0%)	152 (91.0%)	11.454	.001
No	210	46 (21.9%)	164 (78.1%)		
Residential situation					
Living alone	73	13 (17.8%)	60 (82.2%)	0.230	.89
With spouse	160	26 (16.3%)	134 (83.8%)		
With children	144	22 (15.3%)	122 (84.7%)		
Childhood famine					
Yes	156	31 (19.9%)	125 (80.1%)	2.674	.10
No	221	30 (13.6%)	191 (86.4%)		
Childhood poverty					
Yes	208	41 (19.7%)	167 (80.3%)	4.266	.039
No	169	20 (11.8%)	149 (88.2%)		
MVPA					
<2 h/d	118	27 (22.9%)	91 (77.1%)	5.687	.017
≥2 h/d	259	34 (13.1%)	225 (86.9%)		
Smoking status					
Yes	81	19 (23.5%)	62 (76.5%)	4.028	.05
No	296	42 (14.2%)	254 (85.8%)		
Drinking					
Yes	39	6 (15.4%)	33 (84.6%)	0.020	.89
No	338	55 (16.3%)	283 (83.7%)		
Taking statins					
Yes	75	5 (6.7%)	70 (93.3%)	6.248	.01
No	302	56 (18.5%)	246 (81.5%)		
Taking sleeping pills					
Yes	86	7 (8.1%)	79 (91.9%)	5.311	.02
No	291	54 (18.6%)	237 (81.4%)		
Parents died young					
Yes	59	17 (28.8%)	42 (71.2%)	8.231	.004
No	318	44 (13.8%)	274 (86.2%)		

BMI = body mass index, HIV = human immunodeficiency virus, MVPA = medium to high-intensity physical activity.

**Figure 2.**
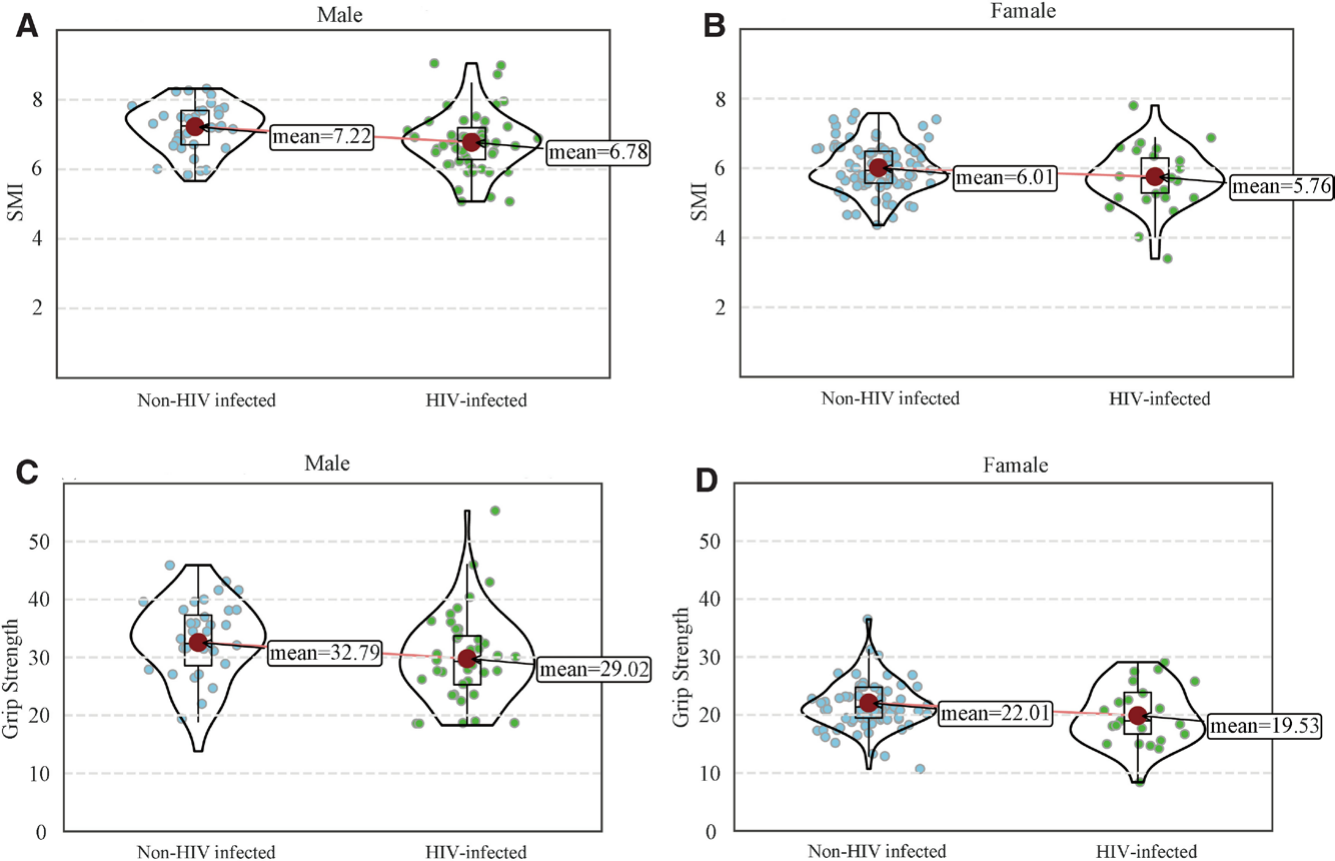
Violin chart of SMI and grip strength in HIV groups by sex. HIV = human immunodeficiency virus, SMI = skeletal muscle index.

Among male elderly, the grip strength for non-HIV-infected individuals was 32.79 ± 7.06 kg, with an SMI of 7.22 ± 0.67 kg/m^2^, while HIV-infected individuals had a grip strength of 29.02 ± 7.29 kg (*t* = −3.329, *P* = .001) and an SMI of 6.78 ± 0.84 kg/m^2^ (*t* = −3.676, *P* < .001). Among female elderly, the grip strength for non-HIV-infected individuals was 22.01 ± 4.00 kg, with an SMI of 6.01 ± 0.70 kg/m^2^, while HIV-infected individual had a grip strength of 19.53 ± 5.09 kg (*t* = −3.075, *P = *.001) with an SMI of 5.76 ± 0.78 kg/m^2^ (*t* = −2.173, *P* = .031) (Fig. 2).

### 3.2. Association between sarcopenia and HIV infection

As shown in Table 1, age, gender, weight, hypertension, diabetes, taking statins, BMI, smoking status, body fat percentage, MVPA, childhood poverty, and parents died young were independent risk factors for sarcopenia (*P* < .05). As shown in Table 2, after adjusting for confounding factors, sarcopenia was associated with HIV infection in elderly in China (*P* = .001). HIV infection group had a higher risk of sarcopenia (odds ratio (OR) = 4.635, 95% confidence interval (CI): 1.920–11.188).

**Table 2:** Association between sarcopenia and HIV infection in elderly in Guizhou province, China.

	β	S.E.	Wald χ^2^	OR	95% CI	*P*
HIV infection	1.534	0.450	11.634	4.635	1.920 to 11.188	.001
Male	3.102	0.614	25.541	22.248	6.680 to 74.097	<.001
Age group	1.605	0.403	15.871	4.976	2.260 to 10.957	<.001
Hypertension	−0.198	0.492	0.162	0.820	0.312 to 2.154	.69
Diabetes	0.128	0.573	0.050	1.137	0.370 to 3.491	.82
Taking statins	−1.012	0.696	2.113	0.364	0.093 to 1.422	.15
BMI	−0.877	0.137	40.964	0.416	0.318 to 0.544	<.001
Body fat rate	0.333	0.061	29.765	1.395	1.238 to 1.572	<.001
Smoking status	−0.831	0.498	2.780	0.436	0.164 to 1.157	.10
MVPA	0.198	0.403	0.242	1.219	0.554 to 2.684	.62
Childhood poverty	−0.485	0.438	1.230	0.616	0.261 to 1.451	.27
Parents died young	0.916	0.473	3.754	2.500	0.989 to 6.317	.05

### 3.3. Association between sarcopenia and HIV infection in sex and age subgroups

As shown in Table 3, After adjusting for confounding factors and conducting stratified analysis, the risk of sarcopenia was significantly increased in the HIV-infected group as compared with the noninfected HIV group in male (OR = 4.466, 95% CI = 1.201–16.610). In female, the association between HIV infection and sarcopenia was also significant (OR = 7.308, 95% CI = 1.726–30.949). In the analysis of age stratification, there was a significant association between sarcopenia and HIV infection in the elderly under 70 years old (OR = 4.477, 95% CI = 1.128–17.762) and over 70 years old (OR = 5.345, 95% CI = 1.130–25.269).

**Table 3:** Stratified analysis of the association between sarcopenia and HIV infection.

	β	S.E.	Wald χ^2^	OR	95% CI	*P*
Male HIV infection^a^	1.496	0.670	4.986	4.466	1.201 to 16.610	.026
Female HIV infection^a^	1.989	0.736	7.295	7.308	1.726 to 30.949	.007
<70 years old HIV infection^b^	1.499	0.703	4.545	4.477	1.128 to 17.762	.033
≥70 years old HIV infection^b^	1.676	0.793	4.472	5.345	1.130 to 25.269	.034

a Adjust for age group, hypertension, diabetes, statin drugs, BMI, smoking status, body fat percentage, MVPA, childhood poverty, and parents died young.

b Adjust for gender, hypertension, diabetes, taking statins, BMI, smoking status, body fat percentage, MVPA, childhood poverty, and parents died young.

## 4. Discussion

This study showed that the prevalence of sarcopenia in the non-HIV infection elderly population of Guizhou Province was 7.8% (21.3% in male and 5.5% in female), while the prevalence was 29.5% (33.3% in male and 13.2% in female) among those infected with HIV. HIV infection was associated with sarcopenia, (OR = 4.635, 95% CI: 1.920–11.188). There is an urgent need to establish a prevention and treatment system for sarcopenia among key populations, with a focus on the muscle health of the elderly HIV-infected population.

Our study found that the sarcopenia situation of the elderly population in Guizhou Province, China, was severe, and the prevalence of sarcopenia cannot be ignored. A meta-analysis utilizing data from 35 articles worldwide involving 58,404 individuals estimated the overall prevalence of sarcopenia in male and female over the age of 60 to be 10% each.^[12]^ Another meta-analysis that included 18,570 individuals showed that the prevalence of sarcopenia in the elderly Chinese community was 12% (95% CI: 10–15%).^[13]^ A Japanese cohort study included 1851 individuals aged 65 and above found the prevalence of sarcopenia was 11.5% in men and 16.7% in women.^[14]^ A cross-sectional survey in Africa found prevalence rates of sarcopenia was 19% in men and 10% in women.^[15]^ In Brazil, a survey by Alexandre et al^[16]^ found prevalence rates of 14.4% in men and 16.1% in women. A study in Korea found that the prevalence rates of sarcopenia in men and women were 11.9% and 6.7%, respectively.^[17]^ The detection rate of sarcopenia in the elderly population of the Yunnan-Guizhou Plateau area in China was reported to be 14.6%, which was similar to the detection rate of this study (13.0%).^[18]^ Studies have indicated that sarcopenia may increase the risk of disability, falls, hospitalization, and death among the elderly.^[19]^ Future investigations with larger, nationally representative samples are needed to assess the prevalence of sarcopenia among the elderly. Current research on risk factors for sarcopenia primarily focused on individuals with diabetes or those who were bedridden for long periods, while few studies paid attention to the potential impact of HIV.

In China, the proportion of elderly individuals with HIV infection was rapidly increasing. In Shanxi Province, the proportion of newly reported cases of HIV among the elderly increased from 7.69% in 2007 to 22.16% in 2018.^[20]^ And in Shaanxi Province, the proportion of elderly HIV cases reported in 2017 was 38.67%.^[21]^ Currently, there was limited research focusing on the muscle health levels of the elderly HIV-infected population. Regarding the association between AIDS and sarcopenia, only a few studies abroad have been reported. A review study, incorporating data from 7 countries worldwide, showed that the prevalence of sarcopenia among individuals with HIV was 23.1%.^[22]^ Compared with HIV-negative matched cohorts (17.0%), individuals with HIV had a 6.1% increased likelihood of developing sarcopenia, a probability that increased with age.^[22]^ A cohort study in Spain indicated that 25.7% of HIV-infected individuals (n = 860) were diagnosed with sarcopenia, the proportion was gradually increased.^[23]^ Furthermore, researches showed that the longer the duration of HIV infection, the greater the risk of developing sarcopenia. In China, research has mainly focused on sarcopenia combined with chronic diseases such as diabetes. To our knowledge, only 1 retrospective study in Hong Kong has reported that among 150 people with HIV, 26 (17.3%) and 41 (27.3%) were classified as having probable sarcopenia according to the AWGS-2014 and AWGS-2019 criteria, respectively. And 27 (18.0%) were confirmed as having sarcopenia as per the AWGS-2019 criteria, but the study did not compare the prevalence to the non-HIV-infected population.^[24]^

Studies have shown that not only the physical fitness of elderly people infected with HIV will decline, but also their immune function will decline due to the attack of HIV virus.^[25]^ This leaded to a significantly higher risk of cardiovascular diseases, skeletal diseases, renal and hepatic dysfunction compared to the general elderly population. An international cohort study showed that the initial outbreak of HIV infection could lead to rapid weight loss and thinness.^[26]^ There are differences in physical condition and lifestyle between HIV infection groups and non-HIV infection groups, due to their ART treatment, poor immune ability and higher psychological stress.^[27]^ ART could lead to a loss of muscle mass and strength. During processes involving immune activation, metabolic disorder, and changes in skeletal muscle tissue protein synthesis and degradation, some antiretroviral pills contribute to key metabolic changes, increasing mitochondrial dysfunction and insulin resistance, thereby promoting inflammation and muscle protein breakdown.^[28]^ This suggests that sarcopenia may have a higher prevalence and lead to more severe adverse outcomes and increased risk of death in individuals with HIV, especially among the elderly.

The findings of this study also suggested that being male, having a low BMI, a low body fat percentage, the lack of moderate to high-intensity physical activity, and exposure to childhood famine were potential risk factors for sarcopenia. Research has shown that a higher BMI was independently associated with a reduced risk of sarcopenia, consistent with this study.^[29]^ Furthermore, physical activity has been shown to potentially reduce the impact of AIDS on sarcopenia and may also reduce inflammation markers in HIV patients, indicating that lifestyle plays a significant role in managing sarcopenia in patients with HIV.^[30]^ A cross-sectional study using data from the China Health and Retirement Longitudinal Study (CHARLS) on 3557 subjects indicated that exposure to famine during childhood may be a risk factor for sarcopenia in the elderly Chinese population.^[31]^ The results were similar to our study.

There is a lack of attention to muscle health in the clinical care and related scientific research of HIV patients now. Moreover, HIV has not been identified as a risk factor in sarcopenia consensus and guidelines.^[22]^ However, sarcopenia may be highly prevalent among individuals with HIV and significantly affect their quality of life and risk of mortality, and this study also suggests that HIV infection is an independent risk factor for sarcopenia.^[32]^ Therefore, policymakers and clinicians should pay attention to the risk and adverse effects of sarcopenia during the HIV diagnosis and treatment process, offering targeted prevention measures, health education, and management services to promote muscle health among HIV patients.

This study is the first to explore the association between sarcopenia prevalence and HIV infection among the elderly population in Guizhou Province, China, using the AWGS-2019 criteria for diagnosing sarcopenia. However, this study has limitations. Being a cross-sectional study, it cannot establish a causal relationship or the underlying mechanisms between sarcopenia and HIV infection. Given that it is unlikely for sarcopenia to cause HIV infection, future research could focus on exploring the biological mechanisms and interventions of their association. Additionally, since the study participants were collected from Guizhou province, this may affect the generalizability of the findings. We also acknowledge the usage of a spring-typed dynamometer in our study as an additional limitation. The difference in dynamometer types, such as the more commonly used Jamar hydraulic or Smedley handgrip dynamometers, may lead to slight variations in measurements. However, research has shown that there was no significant difference in measurement data between hydraulic and spring-type dynamometers for those with a grip strength <35 kg.^[33]^ A wider range of longitudinal studies are needed to further verify the results of this study in the future.

## Acknowledgments

We are grateful to the elderly who took part in the survey.

## Author contributions

**Conceptualization:** Tiecheng Peng, Chen Juhai, Wang Longhang, Yiyi Wang.

**Data curation:** Tiecheng Peng.

**Methodology:** Tiecheng Peng, Xin Jin, Chen Juhai, Yiyi Wang.

**Project administration:** Tiecheng Peng, Xin Jin, Wang Longhang, Yiyi Wang.

**Resources:** Tiecheng Peng, Wang Longhang.

**Software:** Tiecheng Peng.

**Supervision:** Tiecheng Peng, He Juan, Wang Longhang, Yiyi Wang.

**Writing – original draft:** Tiecheng Peng.

**Investigation:** Xin Jin.

**Formal analysis:** Chen Xiong, He Juan, Chen Juhai, Yiyi Wang.

**Validation:** Chen Xiong, Chen Juhai.

**Visualization:** Chen Xiong, Chen Juhai.

**Funding acquisition:** He Juan.

**Writing – review & editing:** Wang Longhang, Yiyi Wang.
